# Small regulatory RNAs: from bench to bedside – a keystone symposia meeting report

**DOI:** 10.1080/15476286.2023.2196046

**Published:** 2023-04-04

**Authors:** Soo Mi Lee, Michael T. Winters, Ivan Martinez, Frank J. Slack

**Affiliations:** a Harvard Medical School Initiative for RNA Medicine, Department of Pathology, Beth Israel Deaconess Medical Center, Harvard Medical School, Boston, MA, USA; b Program in Virology, Division of Medical Sciences, Graduate School of Arts and Sciences, Harvard University, Cambridge, MA, USA; c Department of Microbiology, Immunology, and Cell Biology, West Virginia University, Morgantown, WV, USA; d School of Medicine, West Virginia University Cancer Institute, Morgantown, WV, USA

**Keywords:** Small regulatory RNA, miRNA, siRNA, tRNA, tsRNA, piRNA, sRNA, lncRNA, RNAi

## Abstract

The Keystone Symposium ‘*Small Regulatory RNAs: From Bench to Bedside*’ was held in Santa Fe, New Mexico from May 1–4, 2022. The symposium was organized by Frank J. Slack, Jörg Vogel, Ivan Martinez and Karyn Schmidt, and brought together scientists working in noncoding RNA biology, therapeutics, and technologies to address mechanistic questions about small regulatory RNAs and facilitate translation of these findings into clinical applications. The conference addressed four specific aims: *Aim 1*. Focus on the exciting biology of small regulatory RNAs, highlighting the best current research into the role that small RNAs play in fundamental biological processes; *Aim 2*. Focus on the latest efforts to harness the power of these RNAs as agents in the fight against disease and provide the basic understanding that will drive the invention of powerful clinical tools; *Aim 3*. Attract leaders from both academia and industry working in small RNAs to one place for critical discussions that will advance the field and accelerate the bench to bedside use of this technology; *Aim 4*. Provide a stimulating environment where students, postdoctoral researchers and junior investigators, along with scientists from Biotechnology and Pharmaceutical companies specializing in small regulatory RNAs, can present and discuss their research with the best minds in the field.

## Main text

The Keynote address entitled ‘Viral Noncoding RNAs: New Insights into RNA Structure’ was given by Joan A. Steitz from Yale University. She talked about the Kaposi’s sarcoma-associated herpesvirus (KSHV) noncoding, polyadenylated nuclear (PAN) RNA that is required for late protein synthesis and virus production [1–5]. Remarkably, PAN RNA accumulates to about half a million copies in the nucleus and accounts for about 80% of polyadenylated RNAs in the infected cell [1,4]. This high-level accumulation requires an RNA stabilization element called the ‘element for nuclear expression’ (ENE), which is located near the 3’ end of PAN RNA [6–8], and protects this viral RNA from deadenylation and decay via sequestration of the poly(A) tail [7–9]. ENEs are also found in mRNAs as well as noncoding RNAs [10,11], and they counteract the negative effects of intron loss in transposon mRNAs [12]. She presented the high-resolution crystal structure of a double ENE (dENE) complexed with 28-mer poly(A) to reveal novel modes of interactions between the dENE and poly(A) tail [13]. These findings greatly expand our knowledge of poly(A) tail function in RNA biology.

The first session focused on the role that small RNAs play in cancer. Joshua T. Mendell (University of Texas Southwestern Medical Center) described different modes of microRNA (miRNA)-target interactions and specific post-transcriptional modifications that determine the fate of the target RNA and the Argonaute protein [14,15]. George A. Calin (University of Texas MD Anderson Cancer Center) provided insights into unconventional miRNA functions [16], ultraconservation in human cancers [17,18], and small molecules targeting oncogenic miRNAs [19]. Christine Eischen (Thomas Jefferson University) gave a talk on identifying and characterizing critical miRNAs that target multiple messenger RNAs in one or more cellular pathways in tumour development or suppression [20]. Aurora Esquela-Kerscher (Eastern Virginia Medical School) showed that miR-888 cluster members are promising clinical targets for aggressive prostate cancer [21,22].

The second session highlighted miRNAs in therapeutics. Ivan Martinez (Western Virginia University) showed that SARS-CoV-2 infection specifically reduces cytoplasmic Drosha expression and that alternative splicing of Drosha is regulated differently by distinct SARS-CoV-2 variants. Vishal Patel (University of Texas Southwestern Medical Center) highlighted the promises and challenges of developing anti-miR-17 therapy for autosomal dominant polycystic kidney disease [23]. Raman Bahal (University of Connecticut) gave a talk on targeting miR-21 and miR-10b for glioma therapy by using next generation gamma peptide nucleic acids (γPNAs) and polylactic acid-hyperbranched polyglycerol (PLA-HPG) [24–26]. Murugaiyan Gopal (Harvard Medical School) showed that T-cell intrinsic miR-92a inhibits Treg induction and promotes Th17 differentiation by targeting Foxo1 and that miR-92a silencing represents a promising approach to restore immune tolerance in MS patients by modulating Treg and Th17 responses [27].

The third session examined diverse small regulatory RNAs. Richard I. Gregory (Boston Children’s Hospital/Harvard Medical School) showed that METTL1-mediated m^7^G modification of Arg-TCT tRNA drives oncogenic transformation and that targeting oncogenic tRNA Arg-TCT is a promising anti-cancer strategy [28]. Jörg Vogel (University of Würzburg) provided insights into the promises and challenges of using antisense peptide nucleic acids (PNAs) as species-specific programmable RNA antibiotics for selective targeting of individual bacterial species or genes [29]. Sandra L. Wolin (NCI, National Institutes of Health) showed that damaged tRNAs are signalling molecules that can bind to the CARF (CRISPR-associated Rossman fold) oligonucleotide-binding regulatory domain, leading to activation of an RNA-repair operon [30]. Gisela T. Storz (National Institutes of Health) gave a talk on small regulatory RNAs that are derived within coding sequences and how they function as base-pairing RNAs to regulate protein-coding sequences [31,32]. Daniel Cifuentes (Boston University) showed that the blood-specific miRNA miR-144 regulates chromatin condensation during erythrocyte maturation by down-regulating *HMGN2* expression. Ruben Garcia Martin (Joslin Diabetes Center/Harvard Medical School) provided insights into defining cell-type specific miRNA codes controlling exosomal sorting and cellular retention [33].

The next session focused on small regulatory RNA functions and non-canonical pathways. Shobha Vasudevan (Massachusetts General Hospital) discussed the post-transcriptional regulatory mechanisms involving regulatory noncoding RNAs that contribute to chemoresistance and persistence of quiescent cancer cells. Martin J. Simard (CRCHU de Québec-Université Laval) described the function and regulation of the miRNA-mediated gene silencing pathway during animal development and showed that serine 642 dephosphorylation of Argonaute ALG-1 controls miRNA-induced silencing complex (miRISC) formation [34]. Monika Gullerova (University of Oxford, Wedham College) showed that Dicer dependent tRNA-derived small RNAs (tsRNAs) can mediate nascent RNA silencing in nuclei and discussed the therapeutic potential of novel synthetic tsRNAs-based agents [35]. Elena Piskounova (Weill Cornell Medicine) showed that selenocysteine tRNA methylation regulates stress resistance in melanoma metastasis and outlined the concept of reprogramming the metastatic proteome through tRNA wobble modifications. Soo Mi Lee in the Virology Program at Harvard Medical School, showed that cellular miR-127-3p suppresses KSHV-induced transformation and tumorigenesis via down-regulation of SKP2, identifying the miR-127-3p/SKP2 axis as a viable therapeutic avenue for Kaposi’s sarcoma [36].

The next session examined therapeutic opportunities for small RNAs. Mofang Liu (Shanghai Institutes for Biological Sciences, Chinese Academy of Sciences) showed that PIWI/piwi-interacting RNAs (piRNAs) regulate protein-coding genes in *Drosophila* early embryos and mouse spermatids beyond transposon silencing, and that piRNA 3’ ends are defined by the PIWI-Ins element and contribute to piRNA targeting efficiency [37]. Stefan Engelhardt (Technical University of Munich) provided an overview of miRNA functions in the cardiovascular system and the current state of miRNA-targeted therapeutics and delivery platforms for cardiovascular disease [38]. Karyn Schmidt (Alnylam Pharmaceuticals) gave an overview of Alnylam’s RNAi therapeutics platform and how Alnylam is leveraging a better mechanistic understanding of the RNAi pathway to improve duration, potency, specificity, and delivery of RNAi therapeutics [39,40]. Frank J. Slack (BIDMC Cancer Center/Harvard Medical School) provided an overview of a co-clinical trial with cobomarsen (MRG-106), a miR-155 targeting oligonucleotide, performed with miRagen Therapeutics in patients with diffuse large B-cell lymphoma (DLBCL) and cutaneous T cell lymphoma (CTCL) [41], and described the potential for miRNAs as immune checkpoint inhibitors [42]. John T. Powers (Dell Medical School at the University of Texas at Austin) gave an overview of the mechanisms of *let-7* disruption in neuroblastoma [43] and showed that multiple isoforms of *let-7* agonist H19 long noncoding RNA (lncRNA) are highly expressed in MYCN non-amplified neuroblastoma.

The final session highlighted emerging technologies and novel applications for small RNAs. William J. Greenleaf (Stanford University) described a high-throughput sequencing-based method to identify rules for miRNA target binding and cleavage by Argonaute2 (AGO2) and showed that knockdown can be explained by a biophysical RNAi kinetic model incorporating binding and cleavage rates in live cells [44]. Sabrina R. Leslie (University of British Columbia) described a tether-free, quantitative, single-molecule imaging platform (Convex Lens-induced Confinement (CLiC) microscopy) [45] that enables visualization of RNA interactions under cell-like conditions to help understand and develop RNA therapeutics. loannis S. Vlachos (Beth Israel Deaconess Medical Center) gave an overview of the concept of cancer immunosurveillance and described how single-nucleotide polymorphisms in 3’ UTRs can contribute to the cancer immunoediting process. Byunghee Yoo (Massachusetts General Hospital/Harvard Medical School) showed that RNAi-based PD-L1 inhibitor, MN-siPDL1, is efficacious in highly aggressive models of pancreatic ductal adenocarcinoma with intense desmoplasia [46].

At the end of the last session, Frank J. Slack, Jörg Vogel, Karyn Schmidt, and Ivan Martinez concluded the conference by sharing their thoughts on the growing importance of small noncoding RNAs in medical therapeutics, highlighting the cutting-edge basic and translational research presented at the meeting, with a focus on the inclusion of all kinds of small noncoding RNA species and the diverse range of model systems employed to study them. Importantly, a number of collaborations are already beginning to form – stemming from the conference presentations. Each of the organizers agreed that specific outcomes and promising future directions included: A highlight of the number of advances the field of small noncoding RNAs is currently experiencing; A focus on the connections and collaborations formed from this meeting, and the incorporation of novel techniques into laboratories based on the conference presentations; An emphasis on how the discoveries and therapeutics presented at this meeting are not necessarily islands, but that these discoveries could change how we think about biology and new medicines; A forward focus on what is achievable in the coming years through the discovery of new small RNA species and their implication for our understanding of disease processes and the consequent discovery of novel, RNA-based therapeutics.

The conference offered poster sessions and diverse workshops, which provided an opportunity for researchers to present on emerging topics in the small regulatory RNA field, as well as a career roundtable, which resulted in discussions, guidance and mentoring for early career professionals and facilitated excellent networking opportunities. The emerging topics workshop covered a wide range of topics, including the role of miRNAs in Islet Autoimmunity, miRNA-based anti-tumour therapy, and their role in mitochondrial diseases. The roundtable included sessions from Christine Eischen from Thomas Jefferson University, Stefan Engelhardt from the Technical University of Munich, and John T. Powers from Dell Medical School at the University of Texas at Austin. This represented three different research stages in academia, and they were able to answer specific questions from students – tailoring advice based on student experience and goals.

Overall, conference attendees experienced a set of presentations (from the podium and the posters) at the cutting edge of RNA therapeutics, with a growing appreciation for the potential and challenges that lie ahead. Given the optimal size of the conference, attendees had the opportunity for extensive interactions that will drive innovations in the future. The organizers concluded that the four aims of the conference were achieved.

## Data Availability

Data sharing is not applicable to this article as no new data were created or analysed in this study.

## References

[1] Sun R, Lin SF, Gradoville L, et al. Polyadenylated nuclear RNA encoded by Kaposi sarcoma-associated herpesvirus. Proc Natl Acad Sci, USA. 1996;93(21):11883–11888. 8876232 10.1073/pnas.93.21.11883 PMC38153

[2] Zhong W, Wang H, Herndier B, et al. Restricted expression of Kaposi sarcoma-associated herpesvirus (human herpesvirus 8) genes in Kaposi sarcoma. Proc Natl Acad Sci, USA. 1996;93(13):6641–6646. 8692871 10.1073/pnas.93.13.6641 PMC39079

[3] Zhong W, Ganem D. Characterization of ribonucleoprotein complexes containing an abundant polyadenylated nuclear RNA encoded by Kaposi’s sarcoma-associated herpesvirus (human herpesvirus 8). J Virol. 1997;71(2):1207–1212. 8995643 10.1128/jvi.71.2.1207-1212.1997 PMC191174

[4] Song MJ, Brown HJ, Wu TT, et al. Transcription activation of polyadenylated nuclear rna by rta in human herpesvirus 8/Kaposi’s sarcoma-associated herpesvirus. J Virol. 2001;75(7):3129–3140. 11238840 10.1128/JVI.75.7.3129-3140.2001 PMC114107

[5] Borah S, Darricarrère N, Darnell A, et al. A viral nuclear noncoding RNA binds re-localized poly(a) binding protein and is required for late KSHV gene expression. PLOS Pathog. 2011;7(10):e1002300. 22022268 10.1371/journal.ppat.1002300 PMC3192849

[6] Conrad NK, Steitz JA. A Kaposi’s sarcoma virus RNA element that increases the nuclear abundance of intronless transcripts. Embo J. 2005;24(10):1831–1841. 15861127 10.1038/sj.emboj.7600662 PMC1142595

[7] Conrad NK, Mili S, Marshall EL, et al. Identification of a rapid mammalian deadenylation-dependent decay pathway and its inhibition by a viral RNA element. Mol Cell. 2006;24(6):943–953. 17189195 10.1016/j.molcel.2006.10.029

[8] Conrad NK, Shu MD, Uyhazi KE, et al. Mutational analysis of a viral RNA element that counteracts rapid RNA decay by interaction with the polyadenylate tail. Proc Natl Acad Sci, USA. 2007;104(25):10412–10417. 17563387 10.1073/pnas.0704187104 PMC1965527

[9] Mitton-Fry RM, DeGregorio SJ, Wang J, et al. Poly(a) tail recognition by a viral RNA element through assembly of a triple helix. Science. 2010;330(6008):1244–1247. 21109672 10.1126/science.1195858 PMC3074936

[10] Brown JA, Valenstein ML, Yario TA, et al. Formation of triple-helical structures by the 3’-end sequences of MALAT1 and MENβ noncoding RNAs. Proc Natl Acad Sci, USA. 2012;109(47):19202–19207. 23129630 10.1073/pnas.1217338109 PMC3511071

[11] Wilusz JE, JnBaptiste CK, Lu LY, et al. A triple helix stabilizes the 3’ ends of long noncoding RNAs that lack poly(a) tails. Genes Dev. 2012;26(21):2392–2407. 23073843 10.1101/gad.204438.112 PMC3489998

[12] Tycowski KT, Shu MD, Steitz JA. Myriad triple-helix-forming structures in the Transposable element RNAs of plants and fungi. Cell Rep. 2016;15(6):1266–1276. 27134163 10.1016/j.celrep.2016.04.010 PMC4864102

[13] Torabi SF, Vaidya AT, Tycowski KT, et al. Science. 2021;371(6529):eabe6523. DOI:10.1126/science.abe6523 33414189 PMC9491362

[14] Han J, LaVigne CA, Jones BT, et al. A ubiquitin ligase mediates target-directed microRNA decay independently of tailing and trimming. Science. 2020;370(6523):eabc9546. 33184234 10.1126/science.abc9546 PMC8177725

[15] Han J, Mendell JT. MicroRNA turnover: a tale of tailing, trimming, and targets. Trends Biochem Sci. 2023;48(1):26–39. 35811249 10.1016/j.tibs.2022.06.005 PMC9789169

[16] Dragomir MP, Knutsen E, Ga C. SnapShot: unconventional miRNA Functions. Cell. 2018;174(4):1038. 30096304 10.1016/j.cell.2018.07.040

[17] Calin GA, Liu CG, Ferracin M, et al. Ultraconserved regions encoding ncRnas are altered in human leukemias and carcinomas. Cancer Cell. 2007;12(3):215–229. DOI:10.1016/j.ccr.2007.07.027 17785203

[18] Fabbri M, Girnita L, Varani G, et al. Decrypting noncoding RNA interactions, structures, and functional networks. Genome Res. 2019;29(9):1377–1388. 31434680 10.1101/gr.247239.118 PMC6724670

[19] Winkle M, El-Daly SM, Fabbri M, et al. Noncoding RNA therapeutics – challenges and potential solutions. Nat Rev Drug Discov. 2021;20(8):629–651. 34145432 10.1038/s41573-021-00219-z PMC8212082

[20] Edmonds MD, Boyd KL, Moyo T, et al. MicroRNA-31 initiates lung tumorigenesis and promotes mutant KRAS-driven lung cancer. J Clin Invest. 2016;126(1):349–364. DOI:10.1172/JCI82720 26657862 PMC4701567

[21] Lewis H, Lance R, Troyer D, et al. MiR-888 is an expressed prostatic-secretions-derived microRNA that promotes prostate cell growth and migration. Cell Cycle. 2014;13(2):227–239. 24200968 10.4161/cc.26984 PMC3906240

[22] Hasegawa T, Glavich GJ, Pahuski M, et al. Characterization and evidence of the miR-888 cluster as a novel cancer network in prostate. Mol Cancer Res. 2018;16(4):669–681. 29330297 10.1158/1541-7786.MCR-17-0321 PMC5882523

[23] Lee EC, Valencia T, Allerson C, et al. Discovery and preclinical evaluation of anti-miR-17 oligonucleotide RGLS4326 for the treatment of polycystic kidney disease. Nat Commun. 2019;10(1):4148. DOI:10.1038/s41467-019-11918-y 31515477 PMC6742637

[24] Seo YE, Suh HW, Bahal R, et al. Nanoparticle-mediated intratumoral inhibition of miR-21 for improved survival in glioblastoma. Biomaterials. 2019;201:87–98. 30802686 10.1016/j.biomaterials.2019.02.016 PMC6451656

[25] Dhuri K, Gaddam RR, Vikram A, et al. Therapeutic potential of chemically modified, synthetic, triplex peptide nucleic acid-based Oncomir inhibitors for cancer therapy. Cancer Res. 2021;81(22):5613–5624. 34548334 10.1158/0008-5472.CAN-21-0736 PMC8595710

[26] Malik S, Saltzman WM, Bahal R. Extracellular vesicles mediated exocytosis of antisense peptide nucleic acids. Mol Ther Nucleic Acids. 2021;25:302–315. 34458012 10.1016/j.omtn.2021.07.018 PMC8379631

[27] Fujiwara M, Raheja R, Garo LP, et al. MicroRNA-92a promotes CNS autoimmunity by modulating the regulatory and inflammatory T cell balance. J Clin Invest. 2022;132(10):e155693. DOI:10.1172/JCI155693 35298438 PMC9106347

[28] Orellana EA, Liu Q, Yankova E, et al. METTL1-mediated m^7^G modification of Arg-TCT tRNA drives oncogenic transformation. Mol Cell. 2021;81(16):3323–3338.e14. DOI:10.1016/j.molcel.2021.06.031 34352207 PMC8380730

[29] Vogel J. An RNA biology perspective on species-specific programmable RNA antibiotics. Mol Microbiol. 2020;113(3):550–559. 32185839 10.1111/mmi.14476

[30] Hughes KJ, Chen X, Burroughs AM, et al. An RNA repair operon regulated by damaged tRnas. Cell Rep. 2020;33(12):108527. 33357439 10.1016/j.celrep.2020.108527 PMC7790460

[31] Beisel CL, Storz G. Base pairing small RNAs and their roles in global regulatory networks. FEMS Microbiol Rev. 2010;34(5):866–882. 20662934 10.1111/j.1574-6976.2010.00241.x PMC2920360

[32] Aoyama JJ, Raina M, Zhong A, et al. Dual-function Spot 42 RNA encodes a 15-amino acid protein that regulates the CRP transcription factor. Proc Natl Acad Sci, USA. 2022;119(10):e2119866119. 35239441 10.1073/pnas.2119866119 PMC8916003

[33] Garcia-Martin R, Wang G, Brandão BB, et al. MicroRNA sequence codes for small extracellular vesicle release and cellular retention. Nature. 2022;601(7893):446–451. 34937935 10.1038/s41586-021-04234-3 PMC9035265

[34] Huberdeau MQ, Shah VN, Nahar S, et al. A specific type of Argonaute phosphorylation regulates binding to microRnas during C. elegans development. Cell Rep. 2022;41(11):111822. DOI:10.1016/j.celrep.2022.111822 36516777 PMC10436268

[35] Fazio AD, Schlackow M, Pong SK, et al. Dicer dependent tRNA derived small RNAs promote nascent RNA silencing. Nucleic Acids Res. 2022;50(3):1734–1752. 35048990 10.1093/nar/gkac022 PMC8860591

[36] Lee SM, Kaye KM, Slack FJ. Cellular microRNA-127-3p suppresses oncogenic herpesvirus-induced transformation and tumorigenesis via down-regulation of SKP2. Proc Natl Acad Sci, USA. 2021;118(45):e2105428118. 34725152 10.1073/pnas.2105428118 PMC8609319

[37] Wang X, Ramat A, Simonelig M, et al. Emerging roles and functional mechanisms of PIWI-interacting RNAs. Nat Rev Mol Cell Biol. 2023;24(2):123–141. 36104626 10.1038/s41580-022-00528-0

[38] Laggerbauer B, Engelhardt S. MicroRNAs as therapeutic targets in cardiovascular disease. J Clin Invest. 2022;132(11):e159179. 35642640 10.1172/JCI159179 PMC9151703

[39] Brown CR, Gupta S, Qin J, et al. Investigating the pharmacodynamic durability of GalNAc-siRNA conjugates. Nucleic Acids Res. 2020;48(21):11827–11844. DOI:10.1093/nar/gkaa670 32808038 PMC7708070

[40] McDougall R, Ramsden D, Agarwal S, et al. The nonclinical disposition and pharmacokinetic/pharmacodynamic properties of *N*-Acetylgalactosamine-conjugated small interfering RNA are highly predictable and build confidence in translation to human. Drug Metab Dispos. 2022;50(6):781–797. DOI:10.1124/dmd.121.000428 34154993

[41] Anastasiadou E, Seto AG, Beatty X, et al. Cobomarsen, an Oligonucleotide inhibitor of miR-155, slows DLBCL Tumor cell growth *In Vitro* and *In Vivo*. Clin Cancer Res. 2021;27(4):1139–1149. DOI:10.1158/1078-0432.CCR-20-3139 33208342 PMC8287597

[42] Miliotis C, Slack FJ. MiR-105-5p regulates PD-L1 expression and tumor immunogenicity in gastric cancer. Cancer Lett. 2021;518:115–126. 34098061 10.1016/j.canlet.2021.05.037 PMC8355212

[43] Powers JT, Tsanov KM, Pearson DS, et al. Multiple mechanisms disrupt the let-7 microRNA family in neuroblastoma. Nature. 2016;535(7611):246–251. DOI:10.1038/nature18632 27383785 PMC4947006

[44] Becker WR, Ober-Reynolds B, Jouravleva K, et al. High-throughput analysis reveals rules for target RNA binding and cleavage by AGO2. Mol Cell. 2019;75(4):741–755.e11. 31324449 10.1016/j.molcel.2019.06.012 PMC6823844

[45] Berard DJ, Leslie SR. Miniaturized flow cell with pneumatically-actuated vertical nanoconfinement for single-molecule imaging and manipulation. Biomicrofluidics. 2018;12(5):054107. 30344834 10.1063/1.5052005 PMC6167230

[46] Yoo B, Jordan VC, Sheedy P, et al. RNAi-mediated PD-L1 inhibition for pancreatic cancer immunotherapy. Sci Rep. 2019;9(1):4712. 30886310 10.1038/s41598-019-41251-9 PMC6423142

